# Glucosomes: Glycosylated Vesicle‐in‐Vesicle Aggregates in Water from pH‐Responsive Microbial Glycolipid

**DOI:** 10.1002/open.201700101

**Published:** 2017-07-12

**Authors:** Niki Baccile, Patrick Le Griel, Sylvain Prévost, Bernd Everaert, Inge N. A. Van Bogaert, Sophie Roelants, Wim Soetaert

**Affiliations:** ^1^ Sorbonne Universités, UPMC Univ Paris 06, CNRS Collège de France UMR 7574, Chimie de la Matière Condensée de Paris, UMR 7574 75005 Paris France; ^2^ ESRF—The European Synchrotron High Brilliance Beamline ID02 38043 Grenoble France; ^3^ InBio, Department of Biochemical and Microbial Technology, Faculty of Bioscience Engineering Ghent University, Coupure Links 653 9000 Ghent Belgium; ^4^ Bio Base Europe Pilot Plant Rodenhuizekaai 1 9042 Ghent Belgium

**Keywords:** bolaforms, glycolipids, self-assembly, surfactants, vesosomes

## Abstract

Vesicle‐in‐vesicle self‐assembled containers, or vesosomes, are promising alternatives to liposomes because of their possible hierarchical encapsulation and high stability. We report herein the first example of sugar‐based vesicles‐in‐vesicles, which we baptize glucosomes. These were prepared by using a natural microbial glycolipid (branched C22 sophorolipid) extracted from the culture medium of the yeast *Pseudohyphozyma bogoriensis*. Glucosomes spontaneously formed in water between pH 6 and pH 4 at room temperature, without the requirement of any additive. By means of pH‐resolved in situ small angle X‐ray scattering, we provided direct evidence for the vesicle‐formation mechanism. Statistical treatment of the vesicle radii distribution measured by cryo‐tansmission electron microscopy by using a derived form of the Helfrich bending free‐energy expression provided an order of magnitude for the effective bending constant (the sum of the curvature and the saddle‐splay moduli) of the lipid membrane to *K=*(0.4±0.1) *k*_B_ *T*. This value is in agreement with the bending constant measured for hydrocarbon‐based vesicles membranes.

## 1 Introduction

Liposomes are well‐known supramolecular systems classically obtained from phospholipid mixtures. Sized between 20 nm and several microns and obtained through a series of well‐known methods (e.g. thin‐film hydration, solvent injection, and reverse‐phase evaporation), they are largely studied for their ability to load a given cargo, molecule, or nanoparticle for therapeutic purposes and for their interest in artificial cell design and analytical science, just to cite some.^1^, ^2^ Unfortunately, classical liposomes have one major drawback that concerns their stability throughout the delivery process and leaching of the cargo due to destabilization or degradation of the bilayer membrane by the action of enzymes, such as phospholipase A2.^3^ To overcome such problems and to make more stable liposomes, several new technologies have been studied, and these include, among others, the development of vesosomes (liposomes‐in‐liposomes or multicompartment liposomes),^1^, ^3^, ^4^, ^5^ polymersomes (polymer‐based capsules),^6^ capsosomes (liposome‐containing polyelectrolyte capsules),^7^ proteinosomes (multicompartment protein–polymer conjugates),^8^ and a variety of stimuli‐responsive vesicles^1^ based on lipids, polymers, and their mixtures.

Vesosomes are an interesting class of vesicles used in drug delivery,^9^ and they refer to a multicompartment liposome obtained by using a number of encapsulation strategies. In the first one, spiral folded cochleate cylinders composed of negatively charged dioleyl phospholipids in the presence of calcium ions are mixed with preformed liposomes; upon removal of Ca^2+^ by using ethylenediaminetetracetic acid (EDTA), the cylinders unroll and encapsulate the smaller liposomes.^4^, ^10^ In the second one,^11^ interdigitated lipid bilayers (ILBs) are formed by adding 3 m ethanol to a small unilamellar vesicle solution composed of dipalmitoylphosphatidylcholine (DPPC) below their melting temperature (*T*_m_). The ILBs are then used as precursor membranes to encapsulate a preformed liposomal preparation after heating the solution above *T*_m_. In the third,^12^ multistep approach, cholesterol is used as a binder to “glue” liposomes together and is eventually encapsulated by a hydrated 1,2‐dimyristoyl‐*sn*‐glycero‐3‐phosphocholine (DMPC) cholesterol mixture. In the fourth method,^13^ external compounds such as salts or carbohydrates are used to induce membrane fluctuations in giant vesicles; this results in wobbling of a part of the membrane inward and eventually leads to detachment within the vesicle itself after enclosing external water and forming an inner secondary vesicle. In the fifth method,^14^, ^15^ engineered phospholipid‐based liposomes with complementary surface‐active groups are used to strengthen the interliposomal interaction upon fusion and the possible formation of vesosomes. More recently, microfluidics were used to prepare vesosomes composed of an asolectin/cholesterol mixture.^16^

Although vesosomes have broad application potential and despite the fact that various preformed liposome formulations can be encapsulated, the process is still not easy to handle and has several drawbacks. The mechanism of formation of the external membrane is limited to a few phospholipid types generally containing saturated lipid chains, such as DPPC, because of their ability to form interdigitated bilayers. In most cases, it is even necessary to use additives, such as EDTA, ethanol, or cholesterol, or to heat above the *T*_m_ of the lipid (typical *T*_m_ values are higher than 45 °C, a value which can be too high for some applications). Furthermore, the compositions of the external membrane and the internal lipids are generally not the same, a fact that increases the complexity of the overall formulation, and preformed liposomes, obtained by traditional methods,^2^ must generally be prepared before vesosome formation. Last, but not least, all technologies yield vesosomes with a broad size distribution varying from several hundred of nanometers to several microns, whereas small vesosomes below 200 nm in size, interesting for delivery applications, can only be obtained after extrusion.

In this work, we report for the first time the formation of glycosylated vesosomes, which we call glucosomes, obtained in water at room temperature and near neutral pH (pH 6), by using a pH‐sensitive microbial glycolipid obtained from the yeast *Pseudohyphozyma bogoriensis*. Stimuli‐responsive vesicles are of large scientific and medical interest due to the possibility to use one, or a combination of, external stimuli (temperature, pH, magnetic fields, redox potential, light) to trigger the release of a cargo in biomedical applications.^1^, ^17^ Although the literature is rich in specifically pH‐responsive phospholipid and fatty‐acid vesicles,^18^, ^19^, ^20^, ^21^, ^22^ this work illustrates the first example of a stimuli‐triggered vesicle‐in‐vesicle system.

**Figure 1:**
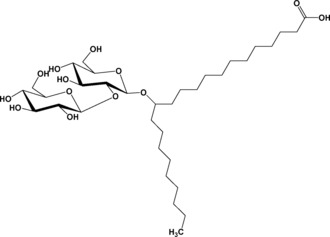
Chemical formula of aSL**‐**C22:0_13_, a branched sophorolipid bearing a hydroxy behenic acid (13**‐**hydroxydocosanoic acid).

*P. bogoriensis* produces a branched bolaform lipid composed of a β‐d‐glucose β(1,2) carbohydrate (sophorose) head group covalently linked at the C13 position of behenic acid (C22:0) through a glycosidic bond and a free COOH group at the C1 position of behenic acid itself (Figure 1). Reports on these compounds date back to 1968 when this novel type of sophorolipid was isolated and structurally characterized.^23^ In contrast to the well‐known sophorolipids produced by yeasts of the *Starmerella* clade, these compounds display a branched structure instead of linear structure and do not occur in the lactonic form (i.e. intra‐esterification of the free COOH to the sugar unit). Despite their intriguing structure and hereto‐related properties, application of *P. bogoriensis* sophorolipids is largely neglected, mainly due to the low production titers of 0.5 to 1.0 g L^−1^ compared to the ones mentioned above for which yields of several hundreds of grams per liter can be obtained.^24^ Herein, we report for the first time on the self‐assembly behavior of branched sophorolipids and discuss their potential in biomedical applications.

Using a combination of cryo‐transmission electron microscopy (TEM) and pH‐resolved in situ small‐angle X‐ray scattering (SAXS), we show that this compound behaves as a typical pH‐sensitive microbial glycolipid and is able to form glycosylated vesicles by simply changing the pH in the 6<pH<4 range at room temperature. However, different from other related microbial glycolipids^25^, ^26^ that form single‐ or multiwall vesicles, we observe the formation of glucosomes. If glucosomes and vesosomes are structurally similar, one should note the following: 1) none of the classical compounds employed for vesosomes (e.g. phospholipids, cholesterol) are used, and glucosomes are only composed of β‐d‐glucose β(1,2) (sophorose)‐containing lipids; 2) the compositions of the external and internal membrane vesicles is expected to be the same; 3) we produce a vesicle‐in‐vesicle system by using a simple pH‐change approach instead of any of the known complex methods described in the literature. The glycolipids shown here certainly contribute to enlarging the wide complexity of glycolipid self‐assembly behavior in water;^27^, ^28^, ^29^, ^30^, ^31^, ^32^ in addition, given the importance of carbohydrates in glycobiology and medicine,^33^ this new class of stimuli‐responsive multicompartment glycosylated vesicles could pave the way to the development of complex delivery systems.

## Materials and Methods

### Synthesis of Sophorolipid aSL‐C22:0_13_

The chemical structure of the microbial glycolipid used in this work is shown in Figure 1. The structure represents the sophorose derivative of 13‐hydroxydocosanoic acid (C22 lipid moiety corresponding to behenic acid), in which the glycosidic bond occurs at the C13 position of the fatty acid. We will refer to this compound as aSL‐C22:0_13_, for which a stands for acidic, SL stands for sophorolipid, C22 indicates the length of the fatty acid, 0 is the number of unsaturations, and 13 is the position of the glycosidic bond. This compound was obtained through microbial production by the yeast *Rhodotorula bogoriensis* MUCL 11 796 recently renamed *Pseudohyphozyma bogoriensis* according to a published procedure.^23^ The yeast was grown at 25 °C in medium containing 50 g L^−1^ glucose, 4 g L^−1^ yeast extract, 0.2 g L^−1^ MgSO_4_ **⋅**7 H_2_O, and 1 g L^−1^ KH_2_PO_4_. Inocula in shake flasks were agitated at 200 rpm, and 3–5 % inoculation volume was used for production in a 150 L fermenter (Sartorius). The pH was controlled at 3.5 by NaOH addition, and the stirring and airflow rates were set at 500–800 rpm and 0.5–1 vvm, respectively, to keep the partial pressure of oxygen (*p*O_2_) at 40 % during the entire stationary phase. Discontinuous feeding of glucose was applied to keep the concentration above 30 g L^−1^, and 20 g L^−1^ rapeseed oil was added upon inoculation. The total cultivation time was 133 h. Cells and remaining oil were removed by microfiltration, and SL was extracted with EtOAc (2×). The SL precipitated upon partial evaporation of EtOAc and was recovered by filtration. The sophorolipids obtained by microbial fermentation contained two, one, or no acetylation(s) at the C6 carbon atom of one or the other glucose moiety. To standardize the SL mixture, the acetyl groups were removed by alkaline hydrolysis: the SLs were dispersed in reverse osmosis water, and the solution was brought to pH 12.5 with sodium hydroxide. The pH was monitored and kept constant during hydrolysis. The reaction was followed up by using HPLC with an evaporative light‐scattering detector (ELSD) (Figure S1 in the Supporting Information) and was stopped by acidification (pH 4.5). The final aSL‐C22:0_13_ product was extracted with ethyl acetate and was subsequently freeze dried.

### Sample Preparation

The aSL‐C22:0_13_ glycolipid was dissolved in Milli‐Q‐grade water at room temperature to give a concentration of 5 mg mL^−1^. The pH was increased up to about 11 by adding 5 m NaOH (10–15 μL), and it was eventually decreased by adding microliter amounts of 0.1–1 m HCl until the solution became turbid in the vicinity of pH 6. This procedure was the same as that used for the pH‐dependent study of related sophorolipids^34^ and glycolipids,^25^ which thus makes the results of this work comparable to those of other microbial glycolipids of a similar chemical nature. This process was adapted for analysis in situ by means of small‐angle X‐ray scattering (SAXS), whereas aliquots were extracted at a given pH and were analyzed by using cryogenic transmission electron microscopy.

### Transmission Electron Microscopy Experiments under Cryogenic Conditions (cryo‐TEM)

These experiments were performed with a FEI Tecnai 120 twin microscope operating at 120 kV and equipped with a Gatan Orius CCD numeric camera. The sample holder was a Gatan Cryoholder (Gatan 626DH, Gatan). DigitalMicrograph software was used for image acquisition. Cryofixation was done with a homemade cryo‐fixation device. The solutions were deposited on a glow‐discharged holey carbon coated TEM copper grid (Quantifoil R2/2, Germany). Excess solution was removed, and the grid was immediately plunged into liquid ethane at −180 °C before it was transferred into liquid nitrogen. All grids were kept at liquid‐nitrogen temperature throughout all experiments. Images were handled by using ImageJ software.^35^

### In Situ Small‐Angle X‐ray Scattering (SAXS)

Data were acquired with the high‐brilliance ID02 beamline (*E*=12.46 keV, sample‐to‐detector distance=1 m) at the ESRF synchrotron (Grenoble, France). In situ experiments employed a flow‐through polycarbonate 2 mm capillary connected to the sample‐containing solution at pH 11.6 through a peristaltic pump. The pH was controlled in situ by using a classical KCl pH meter directly in the experimental hutch, which constantly monitored the pH. The pH changes were obtained by using a 0.1 m HCl solution introduced by a motor‐controlled press syringe. Error bars on the experiments were calculated on the basis of the estimated number of photons detected (accounting for the gain and quantum efficiency of the CCD and phosphor layer), assuming Poisson statistics. The noise of the detector was accounted for by comparison with dark current. Data were acquired by using a CCD camera and were integrated azimuthally to obtain a typical *I*(*q*) (*I* is the intensity as a function of the magnitude *q* of the scattering vector) spectrum. Contribution of the solvent (water at pH 11.6) and capillary were measured prior to the experiment and were duly subtracted during the data treatment. Data were corrected for the transmission of the direct beam and were scaled to be in absolute scale. The *q*‐range calibration was made by using a silver behenate standard sample (*d*ref=58.38 Å).

### Fit of SAXS Data: Micelle Model

The SAXS analysis of the data presented here was performed according to Ref. 26. A core–shell ellipsoid of revolution form factor model was chosen to fit the data in the region above *q*>1 nm^−1^ and in the basic pH region, whereas a core–shell bicelle form factor was employed to describe the lipid bilayer membrane. All models used were developed in the SasView 3.0.0 software (CoreShellEllipsoidXT and CoreShellBicelle).^36^ The general equation of the scattering intensity [*I*(*q*)] is given by Equation (1):

(1) $$I(q)=\frac{\mathrm{scale}}{V}{\left(\rho -\rho_{\mathrm{solv}}\right)}^{2}P(q)S(q)+bkg$$

in which scale is the volume fraction, *V* is the volume of the scatterer, *ρ* is the scattering length density (SLD) and is equivalent to the electron density of the object, *ρ*_solv_ is the SLD of the solvent, *P*(*q*) is the form factor of the object, *bkg* is a constant accounting for the background level, and *S*(*q*) is the structure factor. For the purpose of the present work, we assumed a unitary value of *S*(*q*) in the analyzed range of *q* values (*q*>≈1 nm^−1^). The analytical expressions of *P*(*q*) for a core–shell ellipsoid of revolution and CoreShellBicelle models are provided in Ref. 37. In summary, the ellipsoid model, employed in the 12<pH<6 region, is characterized by the equatorial core radius and shell thickness, the core and shell aspect ratios, and the SLDs of the core, shell, and solvent. In the fitting process, the volume fraction (0.5 wt %), core SLD (7.9×10^−4^ nm^−2^), and solvent SLD (9.4×10^−4^ nm^−2^) are fixed. The procedure to estimate the core and solvent SLD was described elsewhere.^38^ The core SLD was estimated on a dry basis of behenic acid. If water penetration could occur,^38^ we assumed this to be negligible to keep the number of independent parameters as low as possible. To control the fit, we also used a series of additional assumptions: the shell SLD was between 10.0 and 12.0×10^−4^ nm^−2^, which are reasonable values for hydrated sophorose;^38^ the equatorial shell thickness was kept below 1.5 nm to be consistent with the values found in previous studies on analogous sophorose‐containing compounds.^25^, ^26^, ^38^

The core–shell bicelle model was used to analyze the 6<pH<4 region. To adapt this model to the analysis of the vesicles bilayers, we used large values of the bicelle radius (fixed), *R*=100 nm, and we fixed the rim size to zero, which thus made it de facto a lipid core–shell bilayer model. As before, the volume fraction (0.5 wt %), core SLD (7.9×10^−4^ nm^−2^), and solvent SLD (9.4×10^−4^ nm^−2^) were fixed. The face thickness, the face SLD, and the length of the bilayer core were optimized on the basis of previous studies on similar compounds.^25^, ^26^ The fit was controlled by assuming, just as above, that the shell SLD was contained in the range of hydrated sophorose, whereas the shell thickness was also below about 1.5 nm. Finally, the evolution of the micelle‐to‐vesicle ratio with pH was evaluated by a linear combination of the two model functions [Eq. (1)] over the entire pH range to fit the data, as shown in Equation (2):

(2) $$I(q{)}_{\mathrm{Tot}}=xI(q{)}_{1}+(1-x)I(q{)}_{2}$$

in which *x*, between 0 and 1, identifies the relative proportion of morphology 1, described by the model function *I*(*q*)_1_, and morphology 2, described by the model function *I*(*q*)_2_. The general expression for *I*(*q*)_1_ and *I*(*q*)_2_ is given in Equation (1), and all assumptions made above are valid. The main difference lies in the expression for the form factor, *P*(*q*): *P*(*q*)_1_ identifies the core–shell ellipsoid model, whereas *P*(*q*)_2_ identifies the core–shell bilayer model. Given that the use of Equation (2) involves the fitting process over a large number of independent variables (≈20), a process introducing many artifacts, we only used it to evaluate *x*, which corresponds to the micelle‐to‐vesicle ratio (*x*→1 stands for micelles only and *x*→0 stands for bilayers only). All other parameters were kept fixed at their optimum values for both micelle and bilayer, as given above.

### HPLC‐ELSD Analysis

HPLC‐ELSD analysis was performed with an Agilent 1260 Infinity equipped with an Agilent Zorbax Eclipse Plus C18 column (4.6×100 mm to 3.5 μm) at 40 °C. A flow rate of 1 mL min^−1^ was applied, and a gradient of two solvents (A: 0.05 % acetic acid, B: acetonitrile) was applied by using the following method: *t*=0 min: 95 % A and 5 % B, *t*=25 min: 5 % A and 95 % B, *t*=27 min: 5 % A and 95 % B, and *t*=30 min: 95 % A and 5 % B. The HPLC‐ELSD data showed a high degree of purity (99.9 %) of the compound, as illustrated by Figure S1.

### LC–MS Analysis

Analysis of aSL‐C22:0_13_ was done with a Thermo LCQ Deca by the use of RP18 solid phase (150×2 mm, Phenomenex Luna) and solvents 1) methanol with 0.1 % formic acid and 2) water with 0.1 % formic acid as gradients. LC–MS analysis was done with a Shimadzu LC‐10‐AD HPLC system (Shimadzu Europe GmbH, Germany) connected to a quadrupole mass spectrometer (Waters, Milford, MA). Molecules were identified by their native molecular masses after ESI (electrospray ionization) without collision. The results were consistent with previous reports.^23^, ^39^

## 2 Results

**Figure 2:**
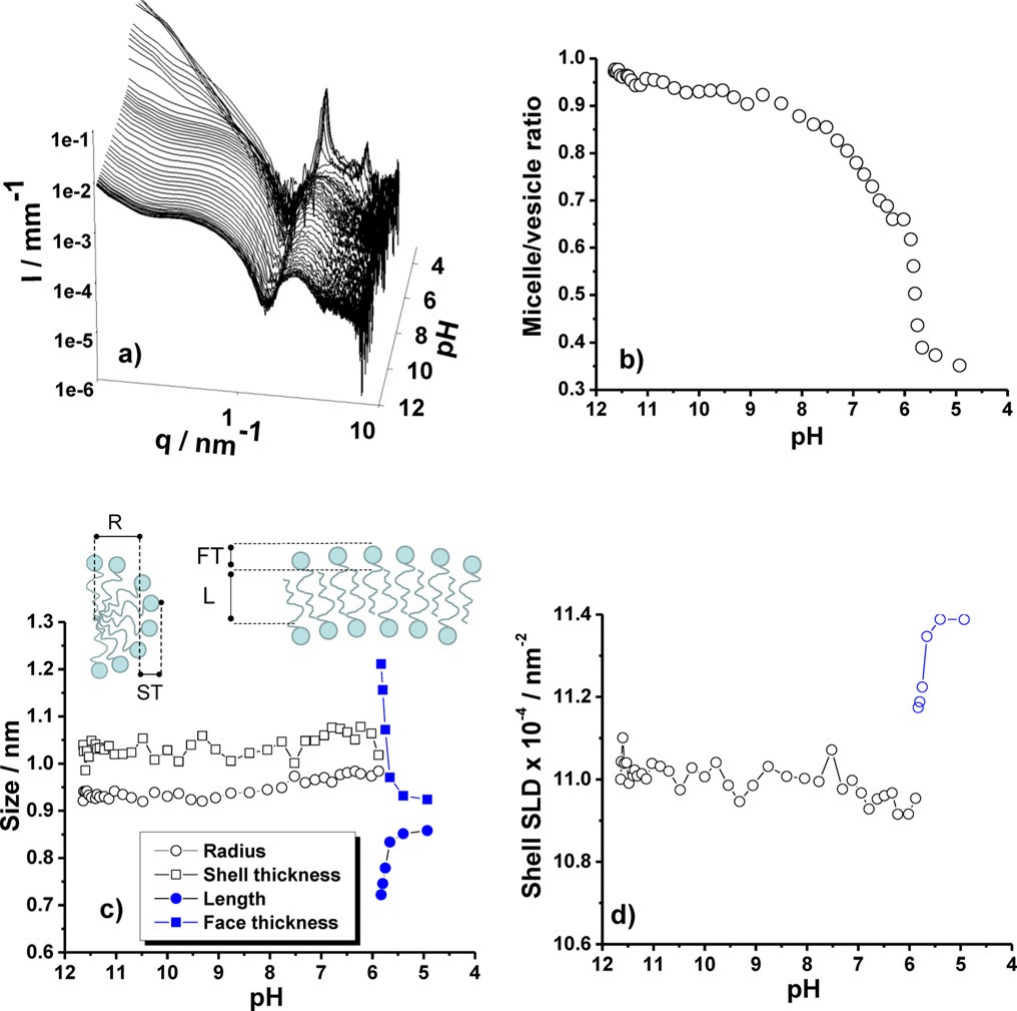
a) pH**‐**resolved in situ SAXS at room temperature on the aSL**‐**C22:0_13_ compound at 0.5 wt % in water showing the characteristic signal of its self**‐**assembly as a function of pH. The evaluation of the micelle**‐**to**‐**vesicle ratio [*x* parameter in Eq. (2)] as a function of pH obtained after fitting the SAXS data [Eq. (2)] is presented in panel b. The pH evolution of the typical micellar radius (R) and hydrophilic shell thickness (ST), as well as the vesicle bilayer length (L) and face thickness (FT), as illustrated by the cartoon, are given in panel c. The micelles (core–shell ellipsoid of revolution) form factor model was used to fit SAXS data in the 12<pH<6 interval, whereas a core–shell bilayer form factor was used to fit SAXS data in the 6<pH<4 interval. The pH evolution of the shell scattering length density (SLD) is presented in panel d. The SLD, which is a parameter of the fit and an equivalent of the electron density of the shell, is useful to identify the hydration/dehydration phenomena of the hydrophilic shell.

**Figure 3:**
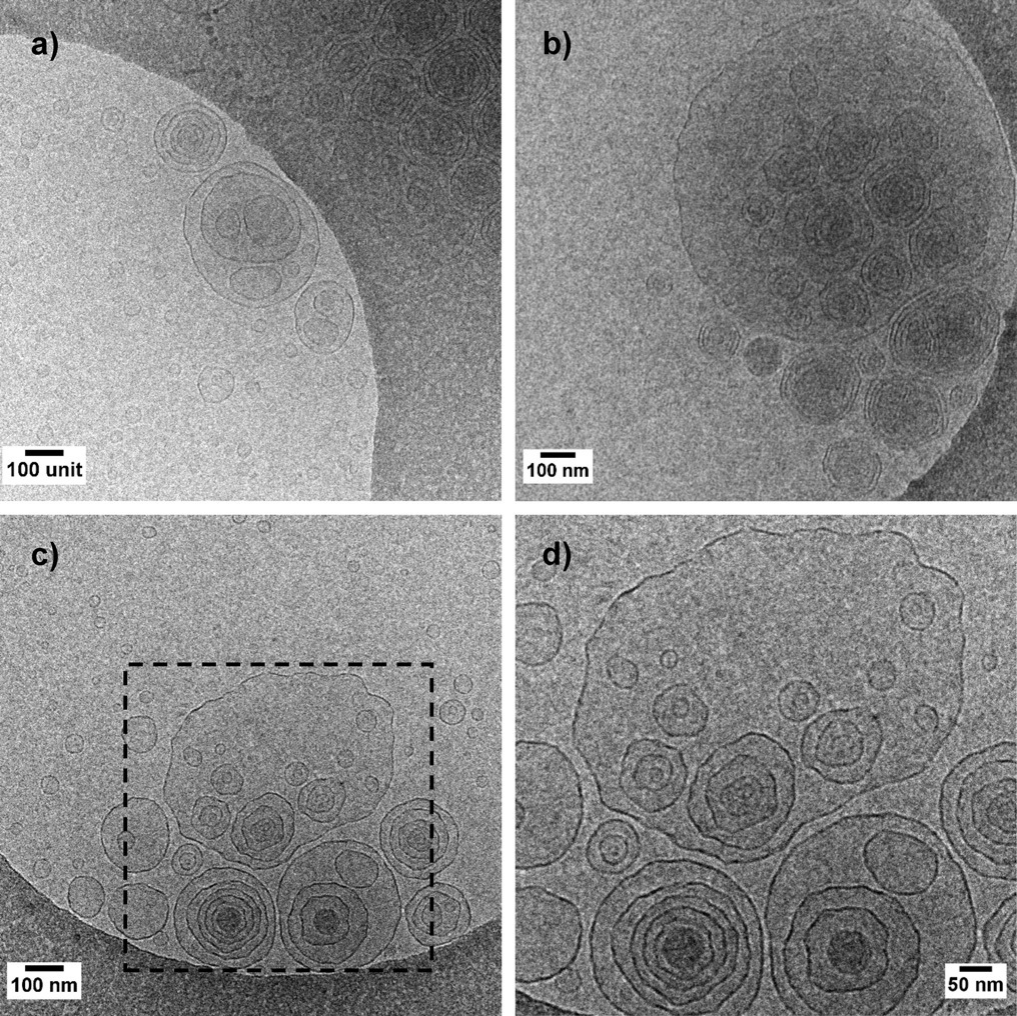
a–c) A series of three cryo**‐**TEM images of glucosomes of aSL‐C22:0_13_ (0.5 w %) recorded at pH 4.9 and room temperature. d) Close‐up image of the black box in panel c. Images show the large number of vesicle‐in‐vesicle systems.

Figure 2 shows the entire set of in situ SAXS data recorded for aSL‐C22:0_13_ between pH 12 and 4. At basic pH, two signals can be identified: below *q*=0.2 nm^−1^ the strong scattering intensity indicates the presence of large objects, whereas above *q*=0.2 nm^−1^ the typical signature of micelles can be identified. Upon lowering the pH, a transition occurs at about pH 6, at which a strong increase in the scattered intensity starts at *q*≈0.9 nm^−1^. Finally, two diffraction peaks at *q*=2.11 and 4.25 nm^−1^, which are indicative of the presence of a long‐range ordered lamellar or onion phase, appear below pH 4. In the 6<pH<4 range, the SAXS signal is characteristic of a lipid bilayer given strong similarities with previous SAXS data collected on microbial glucolipids.^25^, ^26^ The nature of the bilayer was investigated by using cryo‐TEM as a complementary technique. Cryo‐TEM analysis of aSL‐C22:0_13_ in water was recorded in the vicinity of pH 5 to observe the nature of the bilayer structures. Figure 3 shows the massive presence of vesicles throughout the sample holder, and the estimated membrane cross section is (4.0±0.5) nm. The SAXS data could then be fitted by using a core–shell ellipsoid form factor in the micellar region above pH 6 and a bilayer form factor in the 4<pH<6 range until precipitation and formation of a lamellar phase below pH 4; the results from the fit are shown in Figure 2. The micelle‐to‐vesicle ratio throughout the pH jump shows a transition pH at about 6.1±1.0. The typical morphological characteristics of the micelles show a practically constant hydrophobic core radius and hydrophilic shell thickness throughout their stability range; the radius is between 0.90 and 0.95 nm, and the thickness is between 1.00 and 1.05 nm. The hydrophobic core radius is comparable in size to that classically estimated for “classical” acidic sophorolipids and glycolipids;^26^ the shell size is comparable to the size of sophorose and to the experimentally fitted value obtained for acidic C18:1 sophorolipid micelles below pH 6 (≈1.2 nm) but is twice as large as the core radius of acidic C18:1 sophorolipid micelles above pH 7 (≈0.5 nm).^26^ The morphological features of the aSL‐C22:0_13_ micelles seem to be in line with that classically found for microbial glycolipids, whereas possible differences in the hydration layer can explain minor variations (fractions of a nanometer) in the hydrophilic shell thickness.

After the micelle‐to‐vesicle transition pH, the core–shell bilayer model indicates a face thickness of 0.90 nm and a length of 0.85 nm at about pH 5. According to these data, the total vesicle cross section (2×thickness+length) is estimated to be 2.65 nm, a value that is comparable to that obtained by cryo‐TEM, despite a 30 % discrepancy. At the moment, it is unclear whether the bilayer cross‐section is overestimated by cryo‐TEM or underestimated by the fitting process of the SAXS data, as both techniques are subject to experimental error. In all cases, both techniques indicate that the bilayer size is constituted by no more than two aSL‐C22:0_13_ molecules that are most likely interdigitated if one estimates the size of aSL‐C22:0_13_ to be less than 3 nm by using the classical Tanford formula and the length of the hydrocarbon region to be only half of a C22 chain. Figure 2 shows a scheme of the morphological evolution of the self‐assembled structures formed by aSL‐C22:0_13_ against pH.

Finally, the SLD of the shell, which is equivalent to the electron density in the hydrophilic shell region composed of sophorose and water, can be estimated from the SAXS data in the micellar stability domain and its value is constant (11.0×10^−4^ nm^−2^), whereas it slightly increases if vesicles are formed, which is probably indicative of a dehydration process, as already observed for the formation of liposomes from glucolipids.^26^ Analysis of the SAXS data reveals that, as found for saturated microbial glycolipids, the micelles are stable objects until pH 6, as their size and SLD stay practically unchanged. Although it was found that the mechanism of formation of vesicles from pH‐responsive glucolipids followed a classical micelle–cylinder–disk–vesicle mechanism, described for other lipid systems,^40^ it seems that aSL‐C22:0_13_‐based vesicles are formed through a different route that does not include the micelle‐to‐disk transition. In fact, although giant micelles and large disks could be clearly detected for the glucolipids both by cryo‐TEM and SAXS, no hint of their stable presence could be observed in the aSL‐C22:0_13_ system. Considering the sharp, as opposed to smooth, transitions in terms of size and SLD observed between the micellar and vesicle regions, it is likely that the micelles act as reservoirs of matter for the formation of vesicles. Similar arguments were used to describe the micelles‐to‐bilayer formation in microbial glucolipids.^26^

**Figure 4:**
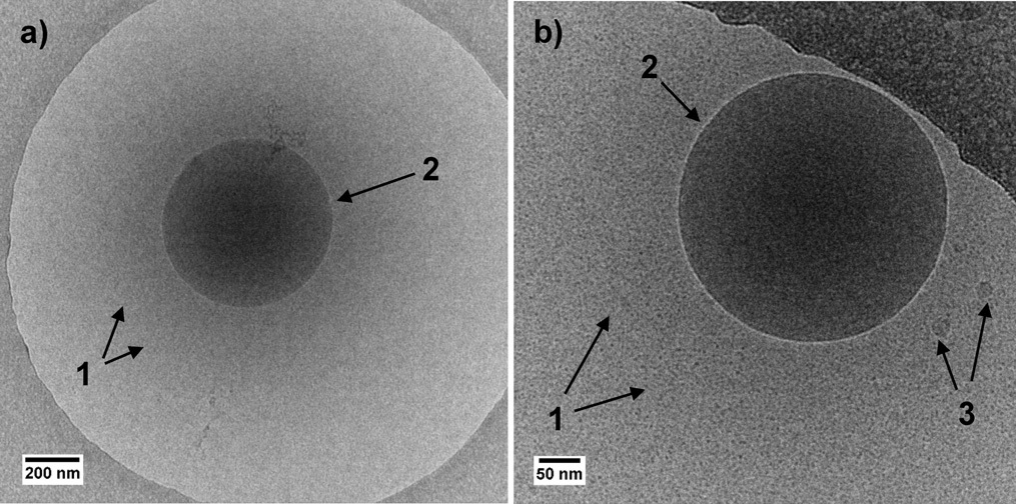
Cryo‐TEM images of glucosomes of aSL‐C22:0_13_ (0.5 w %) recorded at pH 9.4 and room temperature and showing a micellar environment (arrows 1) surrounding vesicular objects of different sizes (arrows 2 and 3). These data agree with the SAXS signal in the basic pH medium.

These assumptions are corroborated by additional cryo‐TEM data recorded at pH 9.4 (Figure 4), in the micellar regime, and at pH 5.9, at the micelle‐to‐vesicle transition. The images show the presence of large micellar aggregates, as indicated by arrows labeled with 1 in Figure 4 a, b. Large (arrows labeled with 2, Figure 4 a, b) and small (arrows labeled with 3, Figure 4 a, b) vesicles are also observed at basic pH in close proximity of the micellar cloud. Very similar images are obtained at pH 5.9, and in no case could micellar fusion into cylinders and disks be observed, as seen for other microbial glucolipids.^26^ Furthermore, the coexistence of (large amounts of) micelles and (few) vesicles at basic pH is neither a surprise nor uncommon. In fact, the corresponding SAXS data have a strong low‐*q* scattering signal, which indicates the presence of spurious amounts of large objects, a fact that is compatible with the vesicles observed by cryo‐TEM. Similar large objects, including rolled sheets and vesicles, were described for glycolipids under basic conditions.^26^ Interestingly, in the transition pH region, cryo‐TEM shows data that is very similar to those presented in Figure 4, that is, an increasing amount of micelles coexisting with micellar aggregates. No filamentous micelles or disks could be observed, which thus corroborates the hypothesis of the reservoir model.

A closer look at Figure 3 taken on the aSL‐C22:0_13_ system at pH 4.9 shows the presence of large vesicles of several hundred nanometers with the embedding of smaller single and multiwalled vesicles ranging from about 25 to 200 nm. Similar systems, called vesosomes, were first reported by the group of Zasadzinski.^10^, ^11^ By analogy, we address the system presented here as glucosomes, referring to a system of vesicles‐in‐vesicles only composed of β‐d‐glucose β(1,2) (sophorose)‐containing lipids. Sophorolipids are a class of microbial glycolipids that commonly refers to C_18_ compounds in their lactonic and acidic forms. It was reported earlier that the sodium salts of acidic sophorolipids formed vesicles in water,^41^ but these data could not be verified, as micelles and nanoscale platelets were reported for the same compound.^42^ Dhasaiyan et al. reported the formation of vesicles by using linolenic acid sophorose lipids,^43^ but scanning electron microscopy (SEM) was employed as the analytical technique, and it was not reported whether the vesicles were single‐walled or if they contained other vesicles. Here, we give direct proof that a sophorose‐containing lipid massively forms vesicles, as corroborated by SAXS, and that the vesicles form a multicompartment system. Nonetheless, despite the structural analogy, glucosomes are phospholipid‐free and are obtained in a single step in water at neutral pH, a fact that makes a big difference with classical vesosomes.^1^, ^10^, ^11^ The latter are generally prepared by embedding a preformed liposomal solution in micron‐scale giant vesicles, either obtained from cochleate cylinders in the presence of Ca^2+^ and EDTA^10^ or from heating lamellar bilayers above the *T*_m_ of the lipid in the presence of ethanol.^11^ The use of cholesterol as a vesicle binder^12^ and the employment of functional vesicles^14^, ^15^ were also reported.

**Figure 5:**
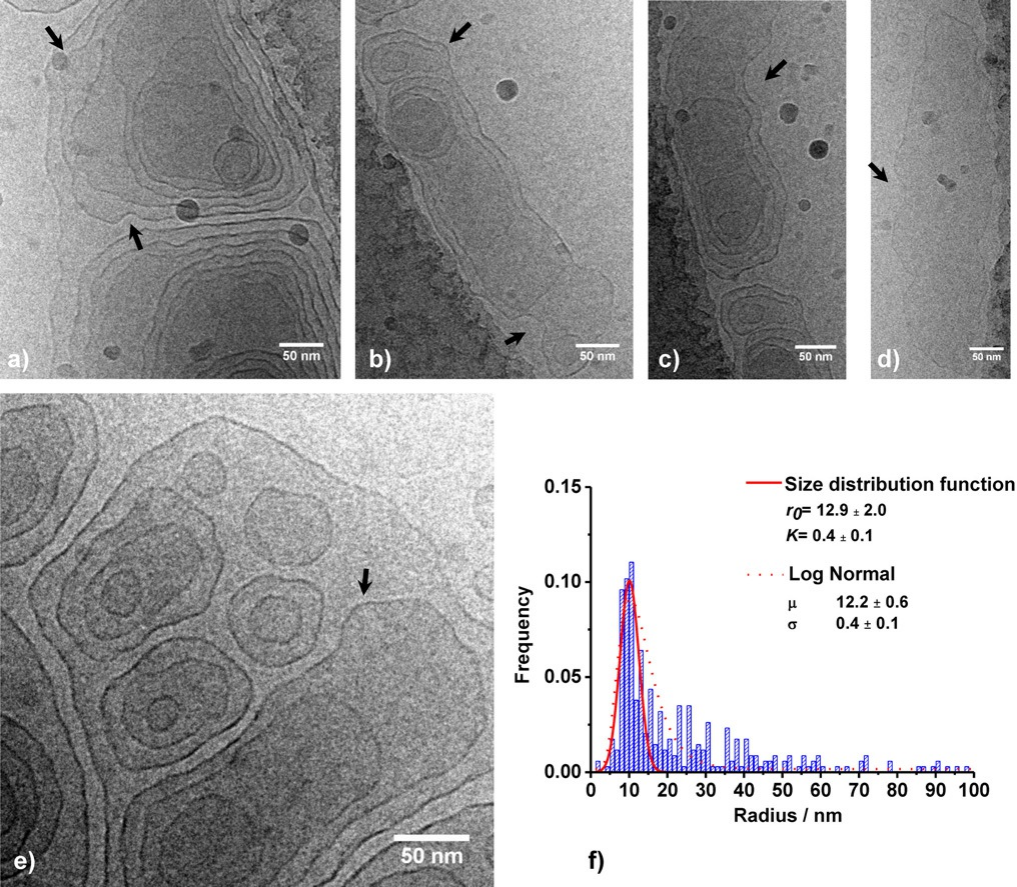
a–e) Series of additional cryo‐TEM images of aSL‐C22:0_13_ (0.5 wt %) recorded at pH 4.9 and room temperature showing the detail of the glucosome surface roughness and structure: arrows point at invagination (negative curvature) and exvagination (positive curvature) sites visible at the membrane surface. f) Distribution (binning size: 2.5 nm; count≈350 vesicles) of the vesicle radii measured from cryo‐TEM images. The data were fitted by using a log‐normal distribution (dotted line) and Equation (4) (continuous line). The *r_0_* value, the mean of the distribution, is defined in Equation (5), whereas *K* refers to the effective bending constant of the vesicle membrane defined in Equation (3).

**Figure 6:**
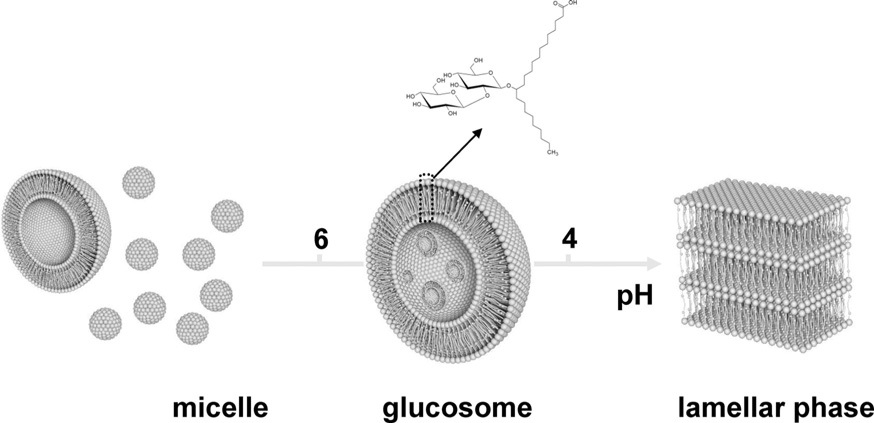
Scheme of the pH‐driven formation of glucosomes by using aSL‐C22:0_13_ branched sophorolipid. According to the in situ SAXS data, the system is composed of a majority of micelles and a minority of vesicles from basic to neutral pH. In the transition pH region, at around pH 6, micelles seem to act as reservoirs of matter for glucosome formation. Below pH 4, a lamellar phase forms.

The glucosomes described in this work strongly differ from these systems in terms of both the composition and formation mechanism. According to the cryo‐TEM and in situ SAXS data, a few vesicles coexist with a large majority of micelles in the basic–neutral pH region (Figure 4); however, pH‐resolved in situ SAXS shows an abrupt micelle‐to‐bilayer transition, which suggests a reservoir‐to‐vesicle‐to‐lamellar^26^ model rather than a micelle‐to‐cylinder‐to‐disks‐to‐vesicle‐to‐lamellar mechanism, as reported for other glucolipids (Figure 6).^25^, ^26^ Although still unclear, upon vesicle formation below pH 6, the glucosome formation mechanism probably goes through membrane wobbling episodes due to spontaneous fluctuations of the surface bilayer, as reported for other systems.^13^ The cryo‐TEM images in our possession seem to confirm this. The arrows in Figure 5 a–e show points at specific wide undulations of the bilayer having a period in the order of tens, or even hundreds, of nanometers, and they can be related to invagination (negative curvature) and exvagination (positive curvature) processes occurring at the membrane surface and are probably at the origin of the glucosome phenomenon. One must also note the enhanced roughness at the vesicle surface (Figure 3 b, d and Figure 5 a–e) compared with the smooth vesicle bilayer observed at basic pH (Figure 4, arrow 2) and where the oscillation period is rather in the order of nanometers. These observations indicate strong flexibility of the membrane at room temperature, a fact that is also reflected by the large polydispersity in terms of vesicle radius. According to the melting temperature of aSL‐C22:0_13_, *T*_m_=58–59 °C,^23^ at room temperature one expects solid‐like behavior of this compound within the membrane, which should then then be stiffer than that observed above. However, the comparison between the actual *T*_m_ and the membrane properties should only be indicative of this class of molecules, as demonstrated by the self‐assembly behavior of acidic subterminal C18:1*cis* sophorolipids, which can form stable crystalline ribbons at room temperature in their pure form,^44^ even though the *T*_m_ of C18:1*cis* (oleic acid) is well‐below room temperature. In contrast, the membranes formed by C18:1*cis* glucolipids are more flexible than those formed by the corresponding C18:0 glucolipids.^25^ The qualitative observations concerning the strong flexibility of the aSL‐C22:0_13_ membrane surface is discussed below.

Figure 5 f reports the radius‐size distribution of the glucosome population (≈350 individual vesicles measured), which varies from a few nanometers up to 300 nm. Even though the histogram is limited to 100 nm for readability purposes, about 5 % of the population has radii in the 100 to 300 nm range. The data can be fitted by using a lognormal distribution (dotted curve) having a mean of *μ*=12.2±0.6 nm and a standard deviation of *σ*=0.4±0.1 nm. Interestingly, it is possible to exploit the radius‐size distribution obtained by cryo‐TEM experiments to determine the effective bending constant of the membrane, *K*,^45^, ^46^ which is defined as [Eq. (3)]:

(3) $$2K=2k+\bar{k}$$

in which *k* and $\bar{k}$ represent the curvature and the saddle‐splay moduli, respectively, and they identify the bending elasticity of the bilayer: *k* is related to the size and $\bar{k}$ is related to the topology. The molar or number fraction, *C_N_*, of vesicles of aggregation number *N* and general radius *R* is related to *K* through [Eq. (4)]:

(4) $$C_{N}={\left\{C_{M}\exp\left[\frac{-8\pi K}{k_{B}T}{\left(1-\frac{r_{0}}{R}\right)}^{2}\right]\right\}}^{\frac{R^{2}}{r_{0}^{2}}}$$

in which *k*_B_ is the Boltzmann constant, *T* is the temperature, and *C_M_* is the molar or number fraction of vesicles of aggregation number *M* and radius *r_0_*, defined as follows [Eq. (5)]:

(5) $$r_{0}=\frac{2k+\bar{k}}{2k}R_{0}$$

and *R*_0_ is defined as the minimum energy, spontaneous, radius of the vesicles. Equation (4) is derived from the Helfrich expression of the bending free energy^47^ by using several approximations, including the assumption that the vesicles are in a metastable state, and if not at equilibrium, they form spontaneously; furthermore, ideal mixing of the vesicles should be verified. The Helfrich expression itself is based on the so‐called harmonic approximation, for which the bilayer thickness (3–4 nm) and the Debye length of ionic surfactants are small relative to the principal radii of curvature of the membrane, *R*_1_ and *R*_2_ (>30 nm).^46^

If *k* and $\bar{k}$ cannot be determined directly, the value of *K* is still useful to characterize the stiffness of a membrane relative to that of other systems: for *K*≈*k*_B_ *T*, the membrane is plastic and thermal undulations generate a broad size distribution, whereas for *K*≫*k*_B_ *T*, the membrane is more rigid than the thermal fluctuations and the size distribution is sharper. Equation (4) can be directly applied to fit the size distribution of the glucosome aSL‐C22:0_13_ system in Figure 5 f (continuous curve), and one finds *r*_0_=(12.9±2.0) nm and *K*=(0.4±0.1) *k*_B_ *T*. The value of *K* below *k*_B_ *T* is in agreement with that expected by a broad size distribution of vesicles, and it can be compared to soft membranes composed of hydrocarbon‐based amphiphiles, such as a mixture of cetyltrimethylammonium bromide (CTAB) and sodium octyl sulfonate (SOS) (0.2<*K*<0.7 *k*_B_ *T* according to the weight ratio) or cetyltrimethylammonium tosylate (CTAT) and sodium dodecylbenzene sulfonate (SDBS).^40^, ^41^ On the contrary, stiffer membranes containing a mixture of hydrocarbons and perfluorinated surfactants can achieve *K* values in the order of 10 *k*_B_ *T*. Despite the nice agreement between our data and the literature, one must be cautious to consider the absolute value of *K*=(0.4±0.1) *k*_B_ *T* as granted. Upon repeating the cryo‐TEM experiment, we systematically found the same type of vesicle‐in‐vesicle system, a fact that gives credit to the data. However, our system is pH dependent, and even if we perform the cryofixation several hours after fixing the pH, the system may still not be at true equilibrium. The membrane cross‐section (2×thickness+length) according to the SAXS data presented in Figure 2 c is about 2.7 nm, a value that is five times lower than the mean value for *r*_0_. From Figure 5, it is clear that Equation (4) does not properly describe the radius distribution, which is better described by the log‐normal distribution. For this reason, the actual value of *K* may be even smaller, and one should probably take the value of *K*=(0.4±0.1) *k*_B_ *T* as the upper limit. Finally, the system under study forms vesicles under acidic pH conditions, and consequently, the sophorolipids are close to neutral, and in this sense, the Debye length condition should be fulfilled.

Figure 6 summarizes the process of glucosome pH formation for the aSL‐C22:0_13_‐branched sophorolipid molecule. We must highlight the fact that stimuli‐responsive vesosomes have so far received little, if no, attention at all.^13^

## 3 Conclusions

In this work, we provided evidence that a branched form of C_22_ sophorolipids, aSL‐C22:0_13_, produced by *Pseudohyphozyma bogoriensis* formed a vesicle‐in‐vesicle colloidal dispersion, which we called glucosomes by analogy with vesosomes, although they were entirely composed of a glycosylated compound. Glucosomes were prepared in water in the 6<pH<4 range at room temperature without the need to add external compounds, as was classically done for the preparation of phospholipid‐based vesosomes. We employed pH‐resolved in situ small‐angle X‐ray scattering (SAXS) by using synchrotron radiation to follow the self‐assembly mechanism of aSL‐C22:0_13_ from basic to acidic pH. The starting system at pH ≈12 was mainly composed of micelles coexisting with a small percentage (<5 %) of large vesicles. This mixture was very stable down to pH ≈6, below which glucosomes were formed. The SAXS data were confirmed by cryo‐transmission electron microscopy (TEM) data recorded at three pH values. Glucosomes were supposed to form from surface fluctuations of the glycolipids bilayer, which we believed to be interdigitated. The bilayer thickness was estimated to be between (4.0±0.5) nm, according to cryo‐TEM measurements, and 2.65 nm, according to modeling the SAXS data at about pH 5; moreover, the length of a aSL‐C22:0_13_ molecule (between COOH and C13, at which branching takes place, and including sophorose) was estimated to be below 3 nm. Employment of a free‐energy‐minimizing mass‐action model in conjunction with the Helfrich bending free‐energy expression allowed estimation of the order of magnitude of the effective bending constant (which is the sum of the curvature and the saddle‐splay moduli) of the lipid membrane to *K*=(0.4±0.1) *k*_B_ *T*. This value well describes the broad radii distribution, and it is coherent with similar hydrocarbon‐based vesicle membranes found in the literature (0.1<*K*<1 *k*_B_ *T*), but its absolute value should be taken as an upper limit for the aSL‐C22:0_13_‐branched sophorolipid glucosome system.

## Conflict of interest

*The authors declare no conflict of interest*.

## Supporting information

As a service to our authors and readers, this journal provides supporting information supplied by the authors. Such materials are peer reviewed and may be re‐organized for online delivery, but are not copy‐edited or typeset. Technical support issues arising from supporting information (other than missing files) should be addressed to the authors.

Supplementary
