# Supplementary material for: Glucosomes: Glycosylated Vesicle‐in‐Vesicle Aggregates in Water from pH‐Responsive Microbial Glycolipid
Source: ChemistryOpen. 2017 Jul 12;6(4):526–33. doi: 10.1002/open.201700101 (PMC5542763; doi:10.1002/open.201700101)

ChemistryOPEN

Supporting Information

© 2017 The Authors. Published by Wiley-VCH Verlag GmbH & Co. KGaA, Weinheim

Glucosomes: Glycosylated Vesicle-in-Vesicle Aggregates in Water from pH-Responsive Microbial Glycolipid

Niki Baccile,*[a] Patrick Le Griel,[a] Sylvain Prévost,[b] Bernd Everaert,[d] Inge N. A. Van Bogaert,[c] Sophie Roelants,[c, d] and Wim Soetaert[c]

open_201700101_sm_miscellaneous_information.pdf

Supporting Information

Figure S 1 - HPLC-ELSD chromatograms of aSL-C22:0$_{13}$ illustrating the compound's purity. The structure was furthermore confirmed by LC-MS analysis (m/z 680).

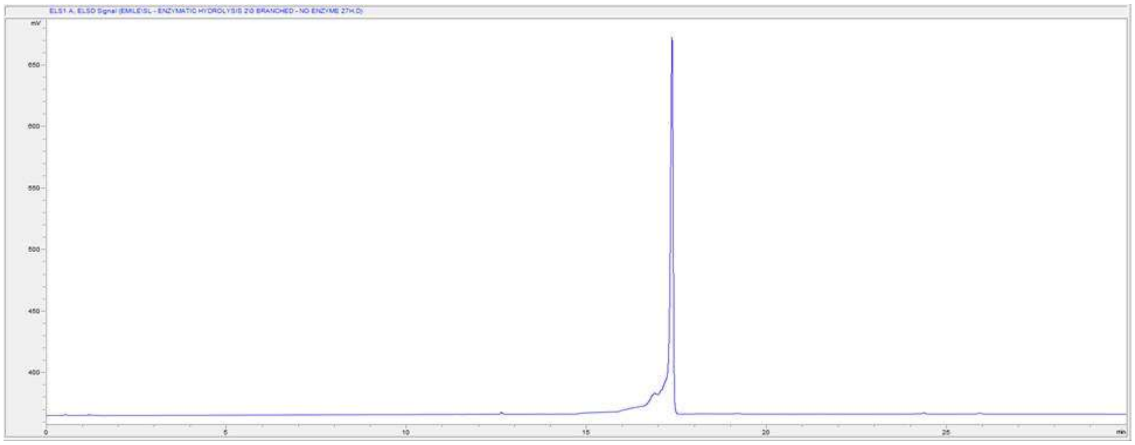

Supplement: Supplementary file 1 — Supplementary [file OPEN-6-526-s001.pdf]
